# On the reliability of highly magnified micrographs for structural analysis in materials science

**DOI:** 10.1038/s41598-020-71682-8

**Published:** 2020-09-07

**Authors:** Martin Wortmann, Ashley Stephen Layland, Natalie Frese, Uwe Kahmann, Timo Grothe, Jan Lukas Storck, Tomasz Blachowicz, Jacek Grzybowski, Bruno Hüsgen, Andrea Ehrmann

**Affiliations:** 1 Faculty of Engineering and Mathematics, Bielefeld University of Applied Sciences, 33619 Bielefeld, Germany; 2 Zentrum für Ultrastrukturelle Diagnostik, 33615 Bielefeld, Germany; 3 Faculty of Physics, Bielefeld University, 33615 Bielefeld, Germany; 4 Institute of Physics – Centre for Science and Education, Silesian University of Technology, 44-100 Gliwice, Poland

**Keywords:** Materials science, Nanoscale materials, Magnetic properties and materials, Nanoparticles, Nanowires, Structural properties

## Abstract

Highly magnified micrographs are part of the majority of publications in materials science and related fields. They are often the basis for discussions and far-reaching conclusions on the nature of the specimen. In many cases, reviewers demand and researchers deliver only the bare minimum of micrographs to substantiate the research hypothesis at hand. In this work, we use heterogeneous poly(acrylonitrile) nanofiber nonwovens with embedded nanoparticles to demonstrate how an insufficient or biased micrograph selection may lead to erroneous conclusions. Different micrographs taken by transmission electron microscopy and helium ion microscopy with sometimes contradictory implications were analyzed and used as a basis for micromagnetic simulations. With this, we try to raise awareness for the possible consequences of cherry-picking for the reliability of scientific literature.

## Introduction

Highly magnified micrographs are the workhorse of materials science. In the majority of all publication in this broad field, they are used to characterize micro- and nanostructures. Usually they are of essential importance for the evaluation of research results up to the point where the entire conclusion of a paper depends on the quality of the selected micrographs. The use of high-resolution microscopy techniques such as electron or ion microscopy and scanning-probe microscopy is nowadays almost a basic requirement for cutting-edge research. It is difficult to overemphasize the importance of these methods for materials science—or medical, biological, chemical and engineering research for that matter. However, it is in the nature of these methods that the smaller the structural area shown, the less information the image contains about how representative these structures are. Depending on the heterogeneity of the sample, confirmation or refutation of a research hypothesis can be only 1 micron apart. Bearing in mind that the selection of micrographs is inherently subjective and unrepeatable, this is particularly problematic when only the bare minimum of micrographs is published and low magnification images are not considered necessary.

Like all humans, scientists are subject to certain biases. Most of us are guided by the basic human desire to show only one’s most presentable accomplishments. On the one hand, scientific work is usually associated with a very busy schedule and, on the other hand, reputation and funding depend largely on the publication history, which is why the first incentive is often to pass through the peer review process as efficiently as possible^1–3^. One way to achieve this may be to pay attention to what is opportune rather than what is representative—i.e. what supports one’s desired hypothesis^4^. The possibility to select the confirming result from a broad range of nano- and microstructures on very heterogeneous samples increases the probability to overinterpretation and overgeneralization of published results. A methodical procedure for the selection of images, as proposed for example by Markey et al. for the selection of fluorescence microscope images of cells^5^, does not seem practicable for materials science, since the topics and intentions behind micrographs are simply too diverse.

However, even under the assumption that micrographs are selected without bias, the significance of very few micrographs of macroscopic samples is often greatly exaggerated. Particularly with regard to a future practical—possibly industrial—application, homogeneity and reproducibility are of essential importance. On the one hand, scientists do not have the time and resources to employ elaborate microscopy methods up to actual statistical significance, and on the other hand, scientific journals are rarely interested in publishing large amounts of similar micrographs. This paradigm leads to a structural problem that contributed to the emergence of the so-called reproducibility crisis^6,7^. Related issues regarding material characterization have been discussed by Linford et al. in the context of X-ray photoelectron spectroscopy^8^ and by Anderson et al. using the example of nanoparticle analysis by transmission electron microscopy (TEM)^9^. However, the scope of these and similar issues does not yet seem to have received the attention that is warranted.

In this study, we explore this problem using the example of electrospun nanofibers—a field of research that has received considerable attention in materials science in recent years^10–13^. In nanofiber research, morphological analysis using sometimes even single micrographs has now become widely accepted^14–23^. TEM images of individual nanofibers from a sample, which usually consists of several million fibers, from which essential statements are generalized, illustrate this issue most clearly. Unexpectedly, this topic has so far hardly been raised in the context of materials science although a large portion of the community seems to be aware of the problem. Most researchers in the field are certainly aware of the extent to which microscopic investigations (of inhomogeneous samples in particular) invite selective reporting and cherry-picking^5^.

Our paper discusses how the selection of micrographs can significantly influence their implications, based on sets of TEM and helium ion microscopy (HIM) images taken on identical samples of poly(acrylonitrile) (PAN) nanofiber nonwovens with embedded nickel-ferrite and magnetite nanoparticles. The different TEM images are then used to demonstrate the potential consequences of a targeted selection of different micrographs using micromagnetic simulations.

## Experimental

The nanofiber nonwovens under examination were produced using the wire-based electrospinning device Nanospider Lab (Elmarco Ltd., Liberec, Czech Republic) with the following spinning parameters: wire-wire voltage 80 kV, nozzle diameter 1.5 mm, carriage speed 100 mm/s, substrate speed 0 mm/s, distance between bottom electrode and substrate 240 mm, distance from ground electrode to substrate 50 mm, temperature 24 °C, and relative humidity 31–32%.

The polymer solution for electrospinning was prepared from 16 wt% polyacrylonitrile (PAN) (X-PAN, Dralon, Dormagen, Germany) dissolved in dimethyl sulfoxide (DMSO) (min 99.9%, S3 chemicals, Bad Oeynhausen, Germany) by stirring for 2 h at room temperature. Magnetic nanoparticles from magnetite (Fe_3_O_4_, Merck, KGaA, Darmstadt, Germany) with particle sizes of 50–100 nm and from diiron nickel tetraoxide (also called nickel ferrite, Fe_2_O_3_/NiO, Merck) with particle sizes < 50 nm were added in a weight ratio of 1:1.8 polymer : nanoparticle. The nanoparticles were dispersed in the solution by manual stirring, followed by ultrasonic treatment for 20 min in an ultrasonic bath at a frequency of 37 kHz, resulting in a temperature increase from 31 to 45 °C.

Investigations of the nanofiber cross-sections were performed with the transmission electron microscope H-500 (Hitachi Ltd, Tokyo, Japan) at 75 kV. Samples were cut to 1 mm × 1 mm, inserted in acetone (min. 99.5%, AppliChem GmbH, Darmstadt, Germany) + Transmit resin (TAAB Laboratories Equipment Ltd, Aldermasten, UK) 1:1 and incubated at room temperature for 20 min, followed by adding 10 drops of Transmit resin, mixing by hand and infiltration in a desiccator for 1 h. After changing the resin, the samples were again infiltrated for 1 h in a desiccator for two times. Afterwards, the samples were polymerized for 12–30 h in a drying chamber (Memmert GmbH + Co. KG, Schwabach, Deutschland) at 70 °C, then pre-cut and finally cut to layers of 50–70 nm using an ultra-microtome (Ultracut, Reichert-Jung, Austria). The slices were contrasted for 5 min with lead citrate (Plano GmbH, Wetzlar, Germany) and uranyl acetate (Science Services GmbH, Munich, Germany).

Additional investigations were done with the helium ion microscope Orion Plus (Carl Zeiss, Jena, Germany) applying an acceleration voltage of 35 kV and a corresponding blanker current of 1.0–1.1 pA. To avoid charging effects during secondary electron detection, a 10 nm gold sputter coating has been applied to the nanofiber nonwovens. Fiber diameters were evaluated using ImageJ 1.51j8 (National Institutes of Health, Bethesda, MD, USA).

Simulations were performed using the micromagnetic solver Magpar, which integrates dynamically the Landau–Lifshitz–Gilbert equation of motion^24^. Simulated structures were evenly distributed magnetite and nickel-ferrite nanoparticles, modelled for a space of 800 nm × 800 nm × 100 nm with the aforementioned diameters and weight ratio of polymer to nanoparticles, as well as larger spheres of 260 nm, corresponding to the identical volumes of magnetic material as in the first simulation. The simulation parameters for magnetite and nickel-ferrite were set as follows: anisotropy constant ${\mathrm{K}}_{1}=-1.10\cdot {10}^{4}\mathrm{ J}/{\mathrm{m}}^{3}$ and ${\mathrm{K}}_{1}=-0.69\cdot {10}^{4}\mathrm{ J}/{\mathrm{m}}^{3}$, respectively; exchange constant $\mathrm{A}=1.2\cdot {10}^{-11}\mathrm{ J}/\mathrm{m}$ in both cases; magnetic polarization at saturation $\mathrm{Js}=0.600\mathrm{ T}$ and $\mathrm{Js}=0.339\mathrm{ T}$, respectively; and Gilbert damping constant $\mathrm{\alpha }=0.01$ in both cases.

## Results

One of the great strengths of electrospinning, which has contributed to its success in recent years, is the ability to process a wide variety of polymers with different additives into nanofibers^25^. Although pure homopolymers usually result in very homogeneous, large-area nonwovens regardless of the type of electrospinning, the addition of further polymers, nanoparticles and other additives leads to increasingly heterogeneous micro and nanostructures^26^. The occurrence of so-called fiber beads, agglomerates, network structures and membranous regions can be observed^27^.

**Figure 1:**
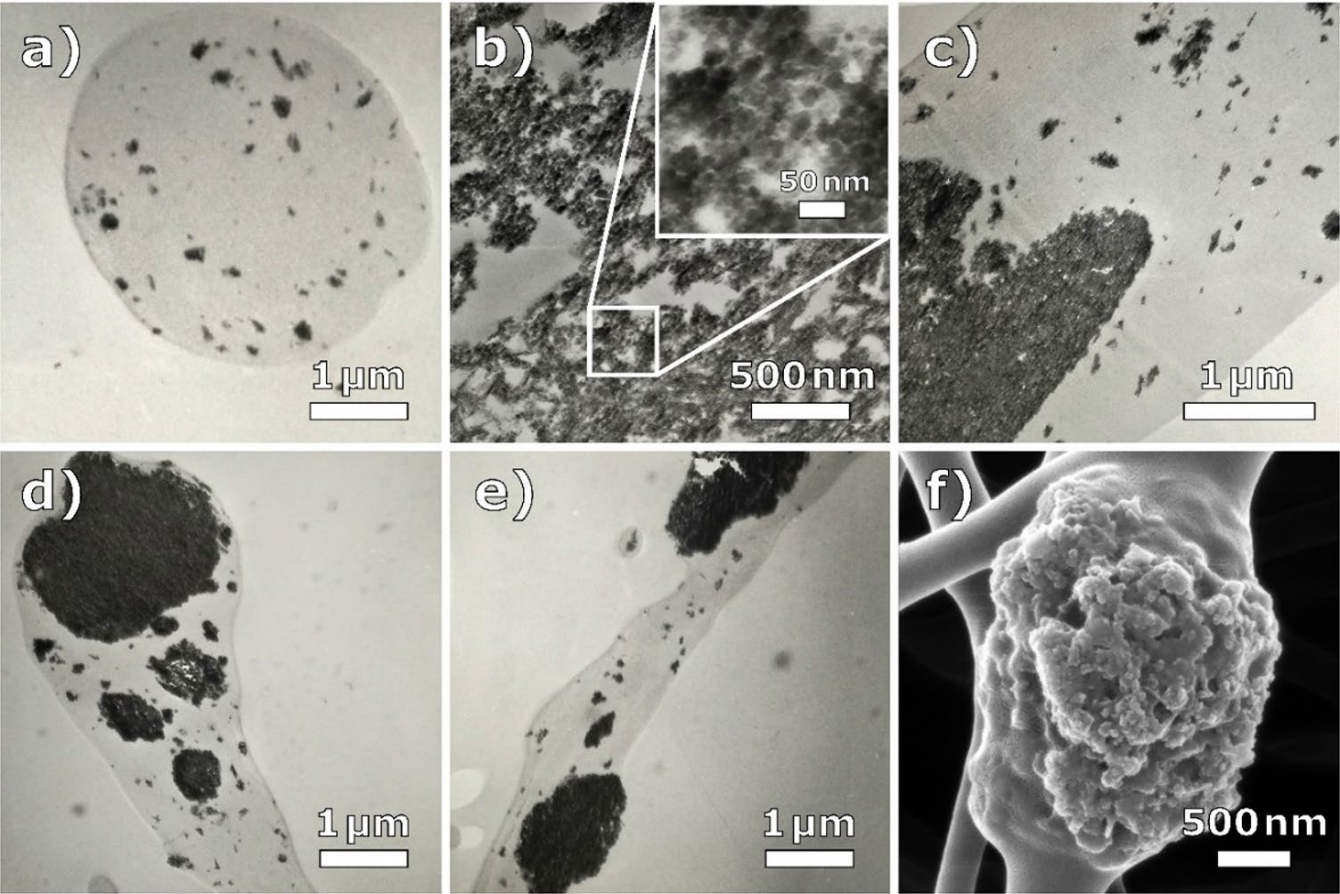
(**a**–**e**) TEM images of individual PAN/nickel ferrite nanofibers; (**f**) highly magnified HIM image from Fig. 3h for comparison.

Figure 1a,b depict TEM images with different magnifications, taken on a PAN/nickel ferrite nanofiber cross-section. From these two images, it could be concluded that the nickel ferrite nanoparticles are distributed relatively evenly along the cross-section of a nanofiber, with small regions of few 100 nm in-between completely lacking nanoparticles.

Figure 1c, however, provides a completely different perspective. Here it is evident that the nanoparticle-free areas between agglomerates can also have dimensions of several micrometers, and the nanoparticles are distributed very asymmetrically along the fiber. Depending on the image chosen for a paper, completely opposed conclusions on nanoparticle distribution in the fibers can be drawn.

Images such as Fig. 1c–e, which depict the cross-section along the axis of symmetry of the fibers, are rarely encountered in journal articles, as they are less aesthetically pleasing, but often contain more or at least important insights. Ignoring such micrographs may deprive an article of crucial information, but on the other hand, it may facilitate the peer review process, as they would most likely be criticized and possibly rejected by reviewers or editors.

For comparison Fig. 1f shows a similar nanofiber from the outside. Here it can be seen that large particle agglomerates are not completely embedded in the polymer and partially burst out of the fiber. Taking a micrograph 5 µm further along the fiber at the same magnification could easily be used to confirm an even particle distribution inside the fiber as seen in Fig. 1a.

**Figure 2:**
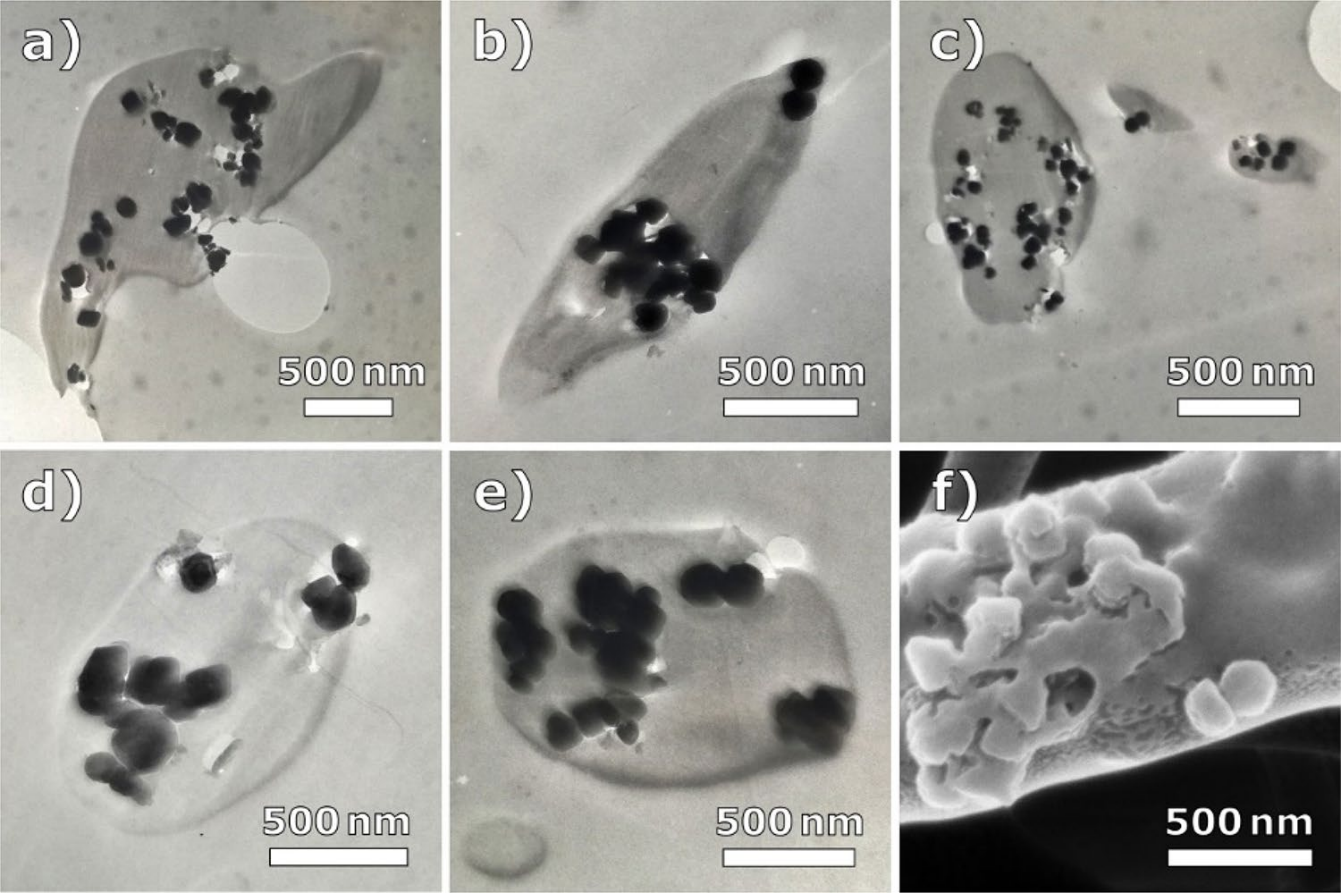
(**a**–**e**) TEM images of individual PAN/magnetite nanofibers; (**f**) highly magnified HIM image from Fig. 4b for comparison.

For PAN nanofibers with embedded magnetite nanoparticles, similar conclusions can be drawn, although here agglomerations are less pronounced. Figure 2 shows a comparison of different nanofiber cross-sections, which could be interpreted either as mostly even distribution of nanoparticles or as a mixture of agglomerates with large particle-free regions in-between, depending on the chosen micrograph. During sample preparation by ultramicrotomy, the relatively large nanoparticles are sometimes displaced. The effect could be misinterpreted as voids or air pockets and is more pronounced in some images than in others. It should be mentioned that the term *even distribution* necessitates a clear definition, e.g. whether the distances between particles inside the fiber are randomly spaced or can be given by a Gaussian distribution with a certain standard deviation; or whether the numbers of particles per matrix volume are in a certain range; or whichever definition is best suited for the respective physical property to be examined.

Again, Fig. 2f shows a HIM image at comparable magnification, indicating that the magnetite nanoparticles can also agglomerate outside the polymeric fiber matrix. Although HIM allows for charge compensation during secondary electron detection by means of an electron flood gun, the samples in this study were sputter-coated with approximately 10 nm of gold, as this is also required for the much more prevalent SEM. The coating thickness is below the average magnetite nanoparticle diameter of 50–100 nm, but still sufficient to allow misinterpretations of their shape (which is rather cubic than round) and to mimic a polymer layer above the nanoparticles. The sputter coating can be clearly distinguished by the icecap-like topology along the sides of the fiber resulting from shadow casting during unidirectional gold deposition.

**Figure 3:**
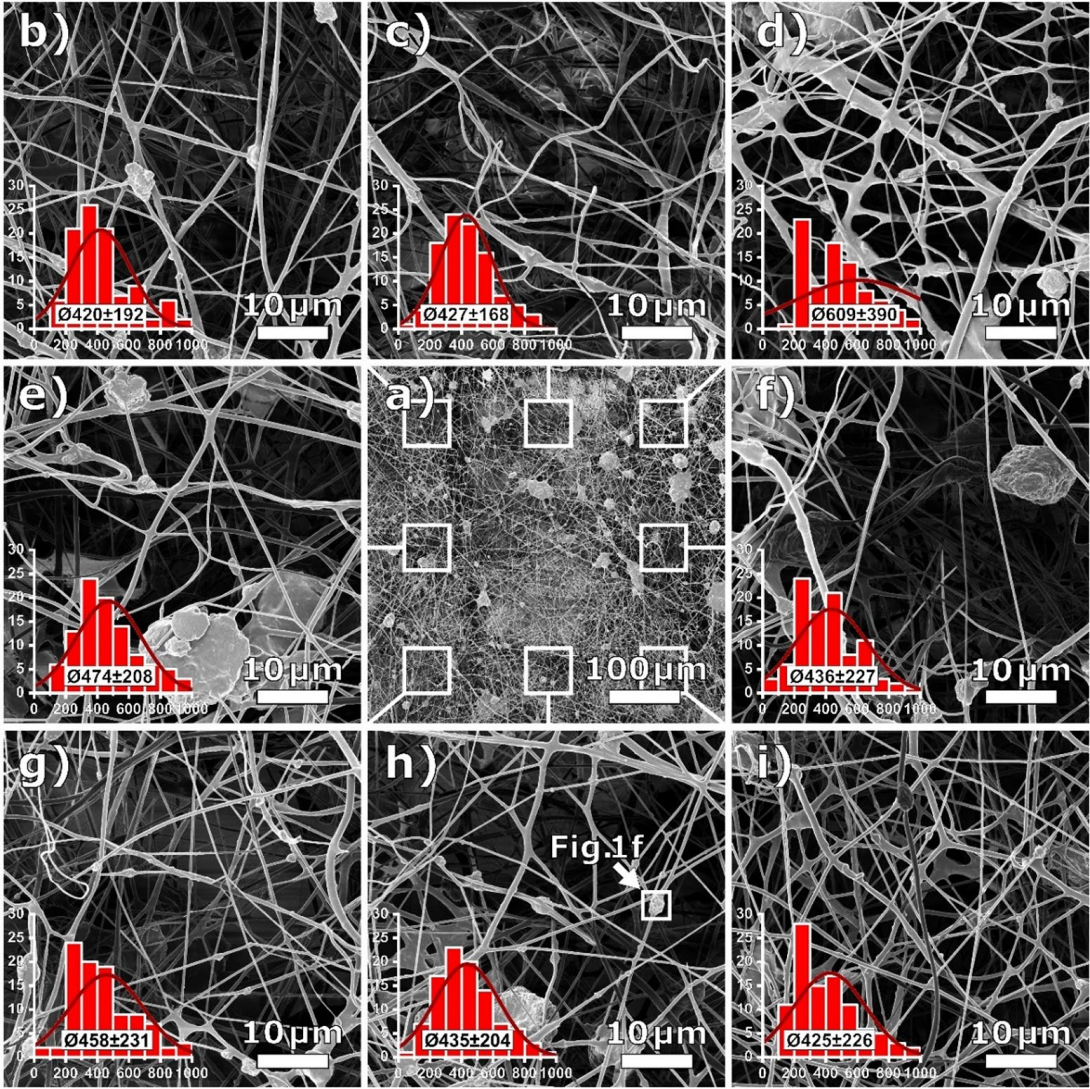
HIM images of a PAN/nickel ferrite nanofiber nonwoven sample: (**a**) overview; (**b**–**i**) magnified images taken at different regions, as defined in a, with inserted fiber diameter histograms, normal distributions and mean fiber diameters in nm. Overall mean fiber diameter is (460 ± 245) nm.

More HIM images depicted in Fig. 3 show the same PAN/nickel ferrite nonwoven from the bird’s eye view. The overview image in Fig. 3a with a field of view of 500 × 500 µm^2^ shows that the nanofiber nonwoven is highly inhomogeneous and brimming with numerous big agglomerates and membranous regions. In order to illustrate the heterogeneity of the nonwoven, Fig. 3b–i show magnified subsections of the overview with a commonly used field of view of 50 µm × 50 µm each. Showing only Fig. 3g or i in a manuscript would suggest the conclusion that the sample consists of only straight uniform nanofibers, possibly with small beads, as often seen in electrospun PAN from a DMSO solution^28,29^. A completely different story, however, is told by Fig. 3e,f,h, clearly showing large agglomerates of polymer and nanoparticles with dimensions of more than 10 µm. The difference between HIM and SEM is particularly noticeable in the outstanding depth of field of the HIM images^30,31^. Several microns can be seen into the nonwoven through the first fiber layers, while deeper lying fibers are still sharply resolved.

These qualitative differences are also reflected in a quantitative analysis, as can be seen from the inserted diameter histograms. Even excluding bigger agglomerates from the diameter evaluation, the average values fluctuate significantly with variable distribution widths. A closer look reveals some differences between typical Gaussian-shaped distributions (Fig. 3c,h) and others, suggesting asymmetric (Fig. 3g,i) or even double-peak distributions (Fig. 3d).

**Figure 4:**
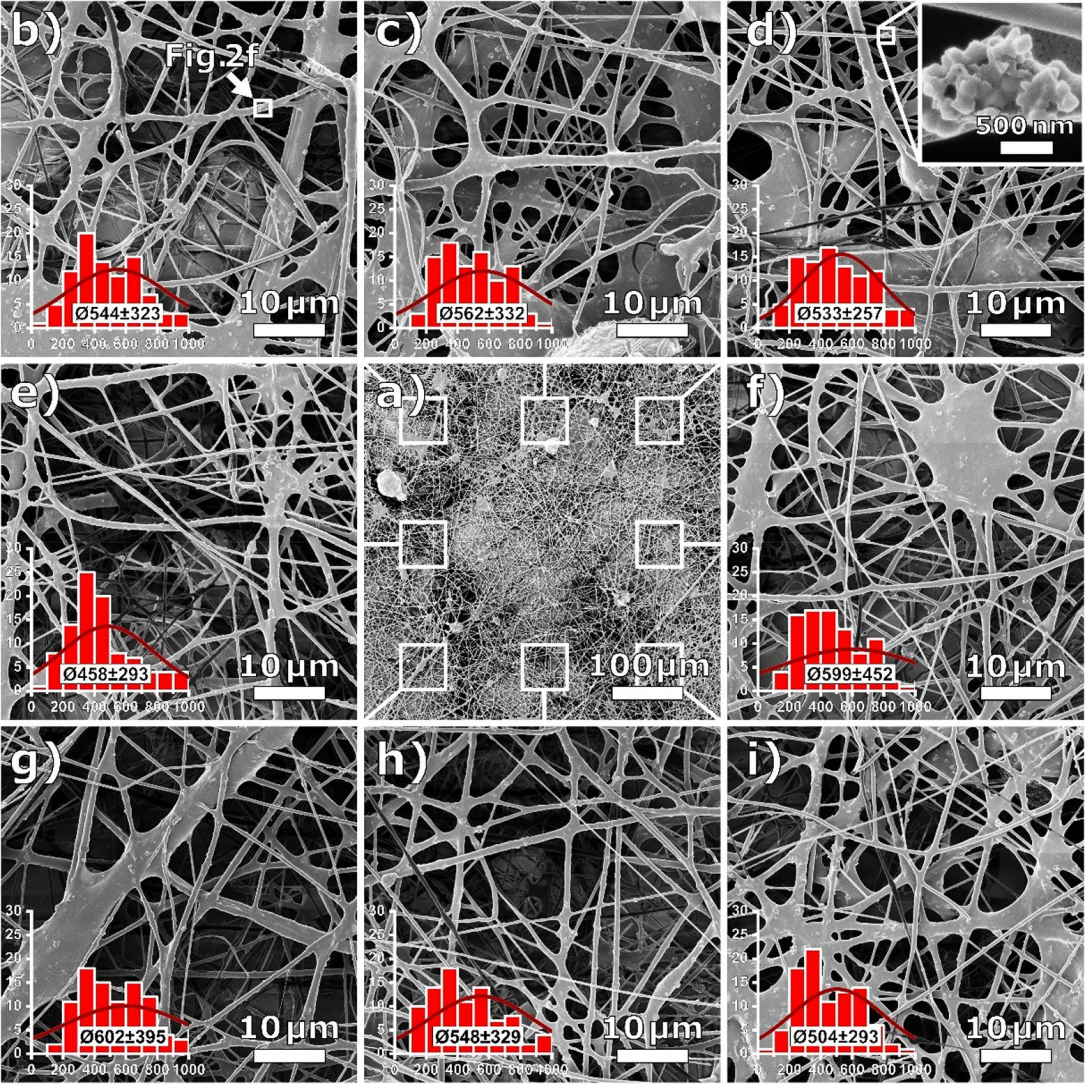
HIM images of a PAN/magnetite nanofiber nonwoven sample: (**a**) overview; (**b**–**i**) magnified images taken at different regions, as defined in a, with inserted fiber diameter histograms, normal distributions and mean fiber diameters in nm. Overall mean fiber diameter is (547 ± 340) nm.

In the same way, HIM images were taken of a PAN/magnetite nonwoven sample, as seen in Fig. 4. Again, some of the magnified images, such as Fig. 4e or h, could unhesitatingly be shown in a manuscript, while others would probably be considered inadequate, such as Fig. 4b,d,f with large membranous regions or Fig. 4g with a disproportionally thick microfiber crossing the image area. As the images show, the samples are so heterogeneous that after a short search with the appropriate magnification, practically any desired hypothesis could be verified. Clearly, TEM micrographs can image a wide range of fiber or membrane cross-sections with any desired nanoparticle arrangement. In this context, it is not even clear whether images like Figs. 1d and 2a show fibers along their longitudinal axis or cross-sections of membranous regions.

While there are many applications for which a heterogeneous structure at the nanometer scale is irrelevant, there are certainly some, where it has a significant influence on the macroscopic properties. Here, this is demonstrated using the example of magnetic properties. These are known to depend strongly on the nanoparticle dimensions, where agglomerates can be expected to show completely different shape anisotropies than individual nanoparticles^32,33^.

**Figure 5:**
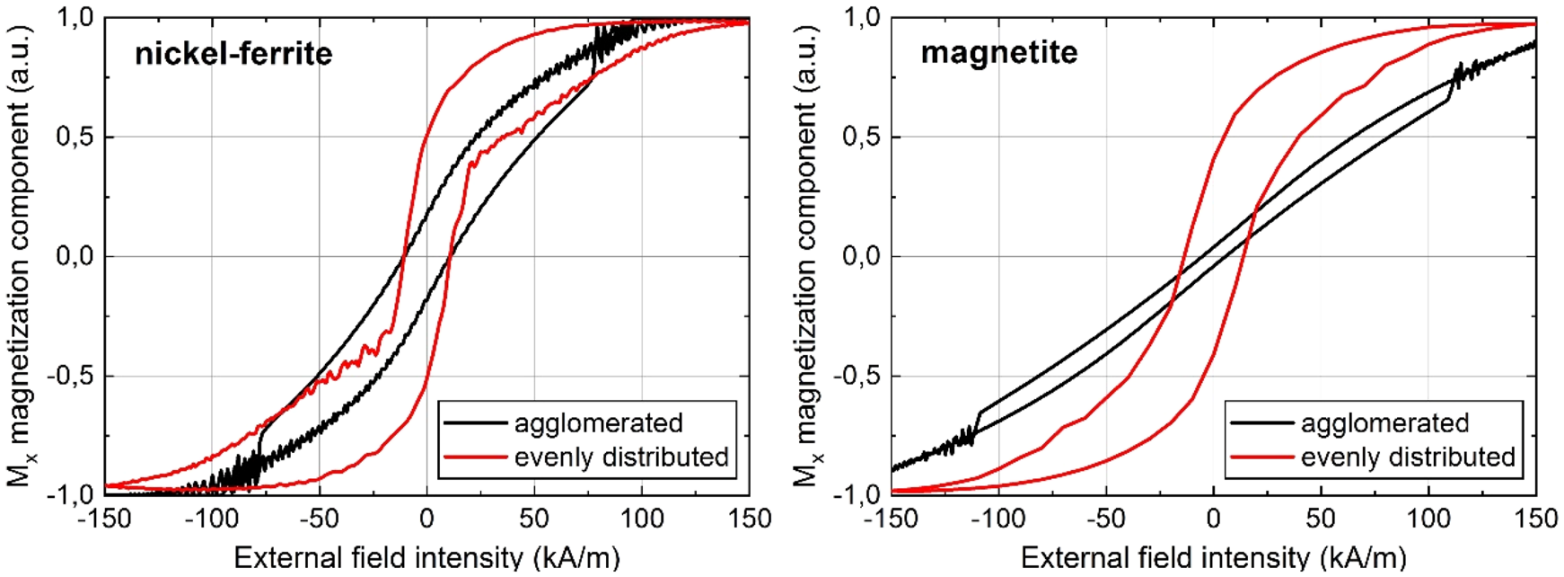
Simulated hysteresis loops for evenly distributed and agglomerated nickel-ferrite and magnetite nanospheres, respectively.

Figure 5 shows hysteresis loops that were simulated based on different nanoparticle arrangements corresponding to different TEM images. Additional simulation results of agglomerated/distributed magnetite nanoparticles, elaborating on the influence of distribution and agglomerate size, can be found in Fig. S2 of the supporting information. As expected, the hysteresis loops show significant differences in terms of the slopes of the curves and, in case of magnetite, even in the coercive fields depending on whether the respective nanoparticles are evenly distributed or agglomerated. The simulations underline that a selective presentation of micrographs has a decisive influence on the predictive power of models based on them.

## Discussion

Nanofibers or nanoparticles are by no means the only fields of materials science that are susceptible to such systemic problems. Nanofibers are used here only as a proxy for practically any kind of micro and nanomaterials, which are evaluated on the basis of highly magnified micrographs. There are of course samples whose relevant structures cannot be displayed at lower magnification (like any atomic structure for example). Both sample preparation and the acquisition of high-resolution images take time and effort and of course, not every evaluation is so significant that it would have to be substantiated by statistical methods. Since a complete uniformity of nanofibers—or any other nanomaterial for that matter—is of little importance for many applications, cherry picking is often only due to the aesthetic demands of humans, anyway. Nevertheless, we, like many others, have already made the experience that published results, be they one’s own, are often unreproducible. The problem, of course, is not only that the way micrographs are published promotes deliberate misconduct, but it leaves readers unaware of the possible reproducibility of the published research. It is rather the exception that low magnification micrographs are provided and only rarely is it stated how many micrographs were taken. It goes without saying that it is not our intention to criticize specific groups. On the contrary, with this we also take a critical look back at our own work.

The bias towards positive outcomes is reflected in many aspects of the peer review process. The publication bias, i.e. the selective publication of positive and disregard for negative results, illustrates the expectations placed on positive outcomes^34^. As has been demonstrated, high-resolution microscopy techniques provide an efficient method of picking the desired confirmation of a research hypothesis from a wide variety of possible image sections. With over 90% of published papers reporting a confirmation of the research hypothesis, materials science is particularly affected by publication bias compared to other natural sciences^35^.

If papers are overly polished and relevant information regarding reproducibility is ignored for fear of being rejected by prestigious journals, the scientific literature will be distorted in the long run^36^. At present, it seems as if the peer review system creates strong incentives to show always only the best and not the most representative micrographs. Researchers are constantly under pressure to publish as frequently as possible with funding often on the line. The way forward leads to more support for honest and sober presentation and evaluation of research results. It is up to all researchers, both as authors and reviewers, to make a small contribution. Our suggestions to improve the situation are thus to both offer and to demand more micrographs to support critical conclusions. Several magnification levels, starting with a large field of view as a reference, can be shown in one image with the highest magnification as insets, allowing significantly more information to be displayed in a slightly larger space. Micrographs, which would otherwise remain unused, can usually be made available as supporting information, which should be encouraged (see Figure S1 of the supporting information). Another way to put the significance of results into perspective is simply to indicate the number of micrographs taken. For samples whose homogeneity and uniformity is explicitly relevant for the core conclusion or proposed application, at least one low magnification image should be mandatory. Ultimately, acknowledging the problem is the first step in solving it.

## Supplementary information

Supplementary Information.

## Data Availability

All data are presented in the main text or the supplementary information.
