# Supplementary material for: On the reliability of highly magnified micrographs for structural analysis in materials science
Source: Sci Rep. 2020 Sep 7;10:14708. doi: 10.1038/s41598-020-71682-8 (PMC7477546; doi:10.1038/s41598-020-71682-8)
Supplement: Supplementary file 1 — Supplementary Information. [file 41598_2020_71682_MOESM1_ESM.docx]

On the reliability of highly magnified micrographs for structural analysis in materials science

Martin Wortmann^1§^, Ashley Stephen Layland^2§^, Natalie Frese^3§^, Uwe Kahmann^2^, Timo Grothe^1^, Jan Lukas Storck^1^, Tomasz Blachowicz^4^, Jacek Grzybowski^4^, Bruno Hüsgen^1^, Andrea Ehrmann^1,^*

^1^ Faculty of Engineering and Mathematics, Bielefeld University of Applied Sciences, 33619 Bielefeld, Germany

^2^ Zentrum für Ultrastrukturelle Diagnostik, 33615 Bielefeld, Germany

^3^ Faculty of Physics, Bielefeld University, 33615 Bielefeld, Germany

^4^ Institute of Physics — Centre for Science and Education, Silesian University of Technology, 44-100 Gliwice, Poland

* Corr. author: andrea.ehrmann@fh-bielefeld.de

^§^ These authors contributed equally to the manuscript.

**Supporting Information**

**
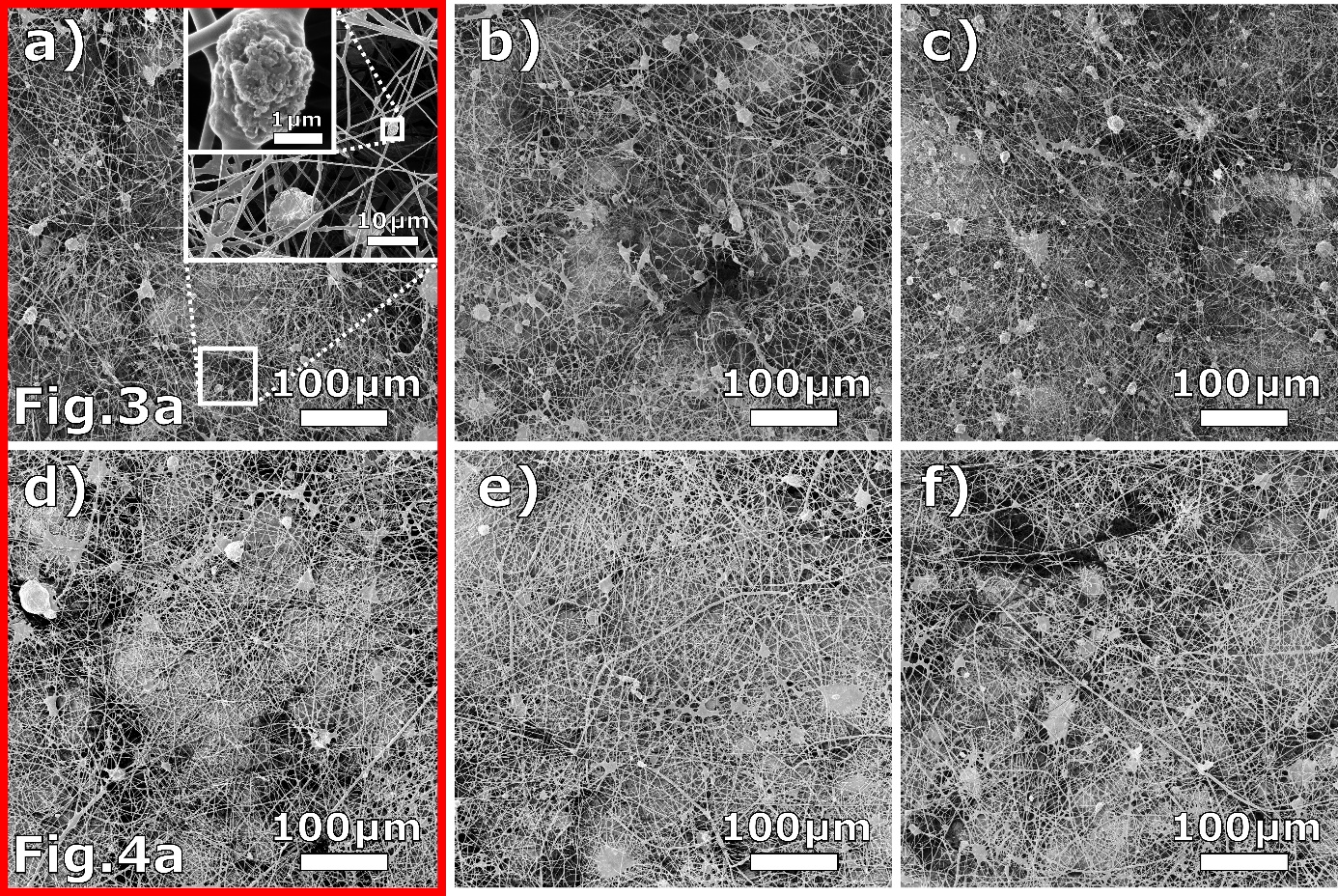
**

**Figure S1.** Additional HIM micrographs of the same (a-c) PAN/nickel ferrite and (d-f) PAN/magnetite nanofiber nonwoven samples, of which a and d have been used for further investigations in the main manuscript.


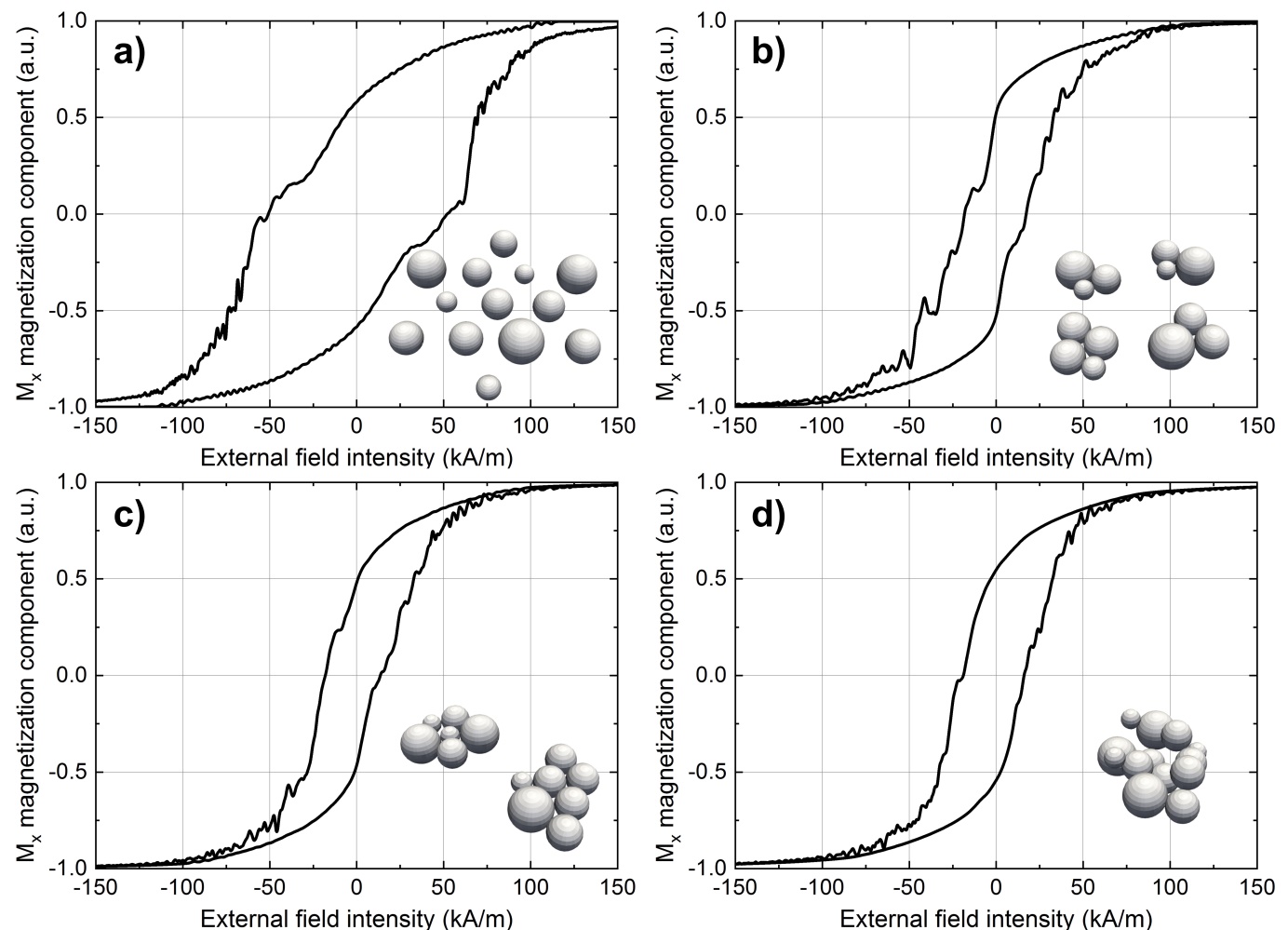


**Figure S2.** Additional simulation results for a set of 13 magnetite nanoparticles with diameters 50 nm (3 x), 75 nm (7 x) and 100 nm (3 x), as suggested by an approximately Gaussian distribution of diameters between 50 nm and 100 nm. The nanoparticles (a) are ideally distributed on a fiber cross-section of thickness 100 nm and diameter 540 nm (i.e. the average value of the nanofiber diameter distributions given in Fig. 4); (b) form agglomerates of 3-4 nanoparticles; (c) form larger agglomerates of 7-8 nanoparticles; (d) fully agglomerated, restricted only by the height of the cross-section of 100 nm. Simulations are performed with the external magnetic field being swept in the plane of the cross-section.
